# Liver HLA-E Expression Is Associated with Severity of Liver Disease in Chronic Hepatitis C

**DOI:** 10.1155/2018/2563563

**Published:** 2018-05-21

**Authors:** Roberta C. Araújo, Fabricio C. Dias, Bruna C. Bertol, Deisy M. Silva, Patrícia H. Almeida, Andreza C. Teixeira, Fernanda F. Souza, Marcia G. Villanova, Leandra N. Z. Ramalho, Eduardo A. Donadi, Ana L. C. Martinelli

**Affiliations:** ^1^Gastroenterology Division, Ribeirão Preto Medical School, University of São Paulo, 14048-900 Ribeirão Preto, SP, Brazil; ^2^Ribeirão Preto Medical School, University of São Paulo, 14048-900 Ribeirão Preto, SP, Brazil; ^3^Pathology Department, Ribeirão Preto Medical School, University of São Paulo, 14048-900 Ribeirão Preto, SP, Brazil; ^4^Immunology Division, Ribeirão Preto Medical School, University of São Paulo, 14048-900 Ribeirão Preto, SP, Brazil

## Abstract

Hepatitis C virus (HCV) can escape from innate and adaptive immunity, making the immune response ineffective. Human leukocyte antigen E (HLA-E) might regulate the antiviral function of immune response and contribute to the persistence of HCV and the severity of liver disease. This study aimed to evaluate the expression of HLA-E in the liver and its association with the severity of liver disease in HCV patients. We performed a retrospective analysis of liver biopsies from 125 HCV patients and from 20 control subjects without liver disease. Liver biopsies were reviewed and classified according to severity of fibrosis and inflammatory activity. The pathologist assessed the magnitude of HLA-E expression in a semiquantitative way, attributing scores from 0 to 3. Immunohistochemistry showed positive for HLA-E in hepatocyte and Kupffer cells. The rate of HLA-E positivity in hepatocytes and Kupffer cells was significantly higher in HCV patients compared to controls. The liver samples classified as severe fibrosis and necroinflammatory activity presented greater expression of HLA-E on Kupffer cells and hepatocytes, with a significant linear association. It indicates that HLA-E expression may have an immunomodulatory effect and a possible role in the severity of liver disease in chronic hepatitis C.

## 1. Introduction

HCV infection is one of the main causes of chronic liver disease worldwide. The long-term impact of HCV infection is highly variable, ranging from minimal histological changes to extensive fibrosis and cirrhosis with or without hepatocellular carcinoma (HCC) [1]. Approximately 80% of patients with HCV infection fail to eradicate the virus and carry a substantial risk to progress towards liver fibrosis/cirrhosis [2]. The exact mechanisms favouring persistent infection and leading to liver fibrosis are not completely known. Numerous studies suggest an association between an impaired immune response and clinical outcome, and there is evidence that immune cells modulate fibrogenesis of hepatitis C [3–5].

Natural killer cells (NK) are specialized lymphocytes that provide a first-line defence through their ability to kill pathogen-infected cells and transformed cells, and they exert an important antifibrotic activity on activated hepatic stellate cells (HSCs) [6–10]. NK cell function is regulated by a balance of inhibitory and activating signals, which are mediated by a diverse array of cell-surface receptors. Inhibitory receptors include a variety of killer cell immunoglobulin-like receptors (KIRs) and C-type-lectin receptors such as CD94/NKG2A. These receptors bind self-major histocompatibility complex (MHC) class I molecules, which are constitutively expressed in normal cells. If the self-class I molecule expression increases in a target cell, it can result in increased inhibitory signals and impaired NK cell activity [11]. Decreased NK cell cytotoxic activity was reported in infections with human cytomegalovirus, Epstein-Barr virus, herpes simplex virus, and HCV. Therefore, altered function of NK cells might be a general mechanism by which viruses escape the immune system [5].

HLA-E is a nonclassical MHC class I molecule, virtually transcribed on all human tissues and cell lines. The role of this molecule in the innate immune response is to present signal sequence-derived peptides of other HLA class I molecules to inhibit NK-mediated cell lysis via recognition by CD94/NKG2A. However, HLA-E can also bind and present other peptide sequences, which can be self or pathogen derived and can be recognized by adaptive T-cells. HLA-E is considered to play a role in both innate and adaptive immunity, via interacting with both NK cells and presenting peptides to antigen-specific CD8+ T-cells [12].

In our study, we analyzed the expression of HLA-E in the liver and its association with the severity of liver disease in chronic hepatitis C patients.

## 2. Material and Methods

### 2.1. Patients

This is a retrospective study conducted through the revision of medical records of 125 patients diagnosed with chronic hepatitis C. All patients were serum HCV RNA positive and had undergone a liver biopsy. Twenty liver biopsies from subjects without liver disease, who died from cardiovascular complications, were used as control. The local ethics committee approved this study (Protocol number: 13812/2014).

### 2.2. Histological Analysis and Immunohistochemistry

The liver samples of the control subjects were reviewed to rule out the presence of inflammatory activity, fibrosis, fat, and iron deposit. The liver biopsies from HCV patients were reviewed and classified according to severity of fibrosis and inflammatory activity (METAVIR classification) [13].

The evaluation of hepatic expression of the HLA-E molecule was performed on liver specimens included in paraffin blocks using the immunohistochemistry technique. In brief, 4 *μ*m-thick slices were made in the paraffin-embedded tissues and mounted on slides pretreated with poly-L-lysine. Antigenic recovery, blocking of endogenous peroxidase, and blocking of nonspecific reactions were performed. The slides were incubated at room temperature in a wet chamber overnight, with the anti-HLA-E antibody MEM-E/02 (EXBIO Antibodies, Czech Republic), at 1 : 100 dilution.

HLA-E expression was classified according to the percentage of HLA-E-positive cells, by manually counting 10 visual fields at ×200 magnification for each liver sample. The results were described in three categories: grade 0 (without positive cell expression), grade 1+ (<25%), 2+ (25–50%), and grade 3+ (>50% of positive cell expression).

### 2.3. Statistical Analysis

Continuous variables were expressed as mean values, standard deviation, and range. Categorical variables were expressed with frequency and percentage. A chi-square analysis and a chi-square for linear trend were used to compare categorical variables, and the continuous variables were analyzed using the Kruskal-Wallis test.

A *P* value less than 0.05 was considered to be significant. Statistical interpretation of data was performed using Statistical Package for Social Sciences (SPSS) version 17.0 for Windows.

## 3. Results

The mean age of HCV patients and control subjects was 49.7 (±11.7) and 58.7 (± 20) years, respectively. Most HCV patients were male (50.8%), and 55% of the control subjects were female. Genotype 1 was the most prevalent (68% of the patients). In HCV-infected samples, the METAVIR classification for liver biopsies showed the following: F0/1: 31%, F2: 33%, F3/F4: 36%, A0/1: 32%, A2: 42%, and A3: 26%. In the control subjects' liver samples, there was no inflammation activity, fibrosis, fat, or iron deposit.

**Figure 1:**
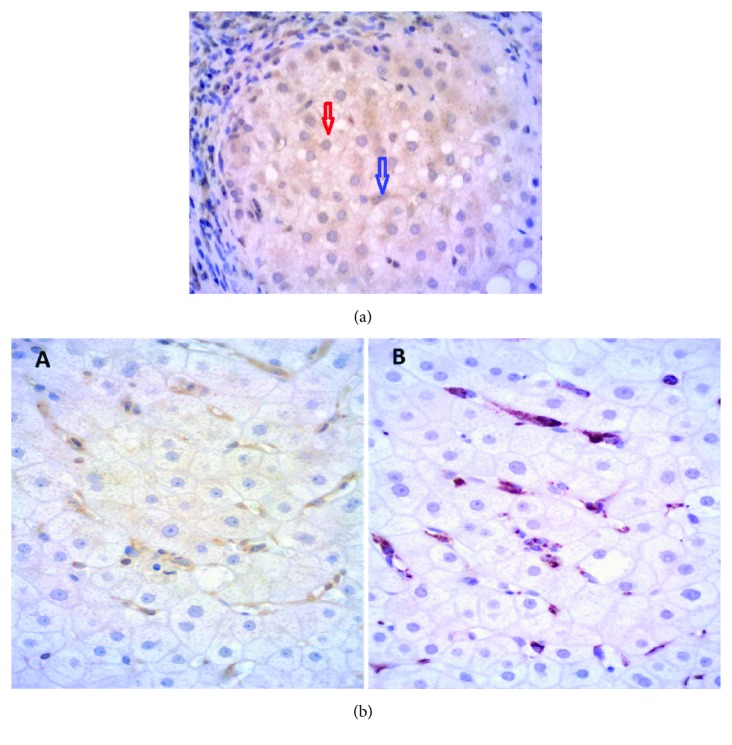
(a) HLA-E positivity in hepatocytes (red arrow) and Kupffer cells (blue arrow). (b) To analyze the identity of intrahepatic HLA-E-positive cells, liver samples were double-labeled for HLA-E and CD68. All sections were immunostained with the indirect immunoperoxidase method and counterstained with hematoxylin. In double-labeling experiments, we incubated the sections with CD68-specific antibodies after performing the HLA-E-specific immunoperoxidase reaction. A: HLA-E-specific immunoperoxidase reaction (yellow); B: anti-CD68 (red) to confirm that these positive cells were Kupffer cells.

Immunohistochemistry showed positive for HLA-E in hepatocyte cytoplasm and Kupffer cells. These cells were identified according to their morphology, and a costaining with CD68-specific antibodies, for Kupffer cells, was done (Figures 1(a) and 1(b)).

The rate of HLA-E positivity in hepatocytes and Kupffer cells was significantly higher in HCV patients compared to controls (58.6% × 20% and 45.3% × 10%, resp.; Table 1).

Patients older than 40 years and the male gender were associated with a higher frequency of HLA-E expression. Sixty-four percent of patients older than 40 years presented HLA-E expression on hepatocytes, compared to 36% of patients aged less than 40 years (*P* = 0.04). Fifty-five percent of males presented HLA-E expression in Kupffer cells, compared to only 34% of female patients (*P* = 0.02).

**Figure 2:**
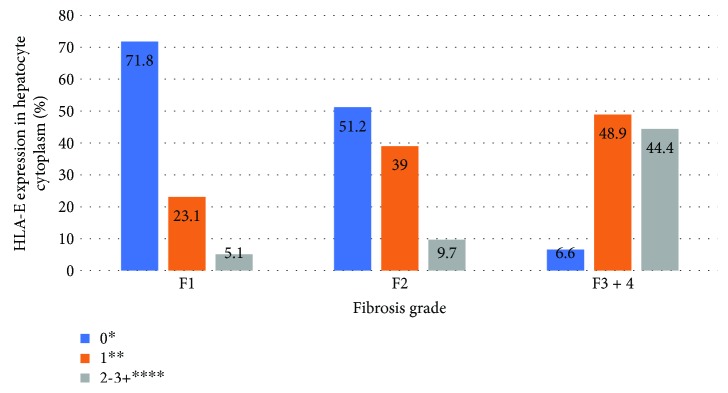
Association between HLA-E expression in hepatocyte cytoplasm and liver fibrosis. HLA-E expression was described in the following grades: 0^∗^ (without positive cell expression), 1+^∗∗^ (<25%), 2-3+^∗∗∗∗^ (>25 or >50% of positive cell expression). Liver fibrosis was described according to the METAVIR classification: F1: portal fibrosis without septa, F2: portal fibrosis with rare septa, F3-4: numerous septa without and with cirrhosis. *P* < 0.001.

**Figure 3:**
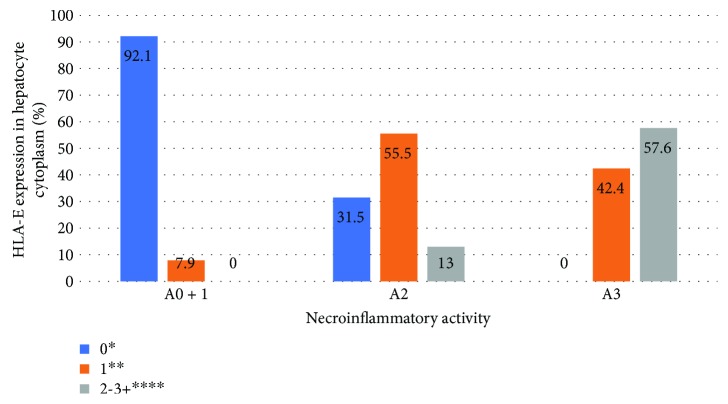
Association between HLA-E expression in hepatocyte cytoplasm and liver necroinflammatory activity. HLA-E expression was described in the following grades: 0^∗^ (without positive cell expression), 1+^∗∗^ (<25%), 2-3+^∗∗∗∗^ (>25 or >50% of positive cell expression). Liver necroinflammatory activity was described according to the METAVIR classification: A0 + 1: no or mild activity, A2: moderate activity, A3: severe activity. *P* < 0.001.

**Figure 4:**
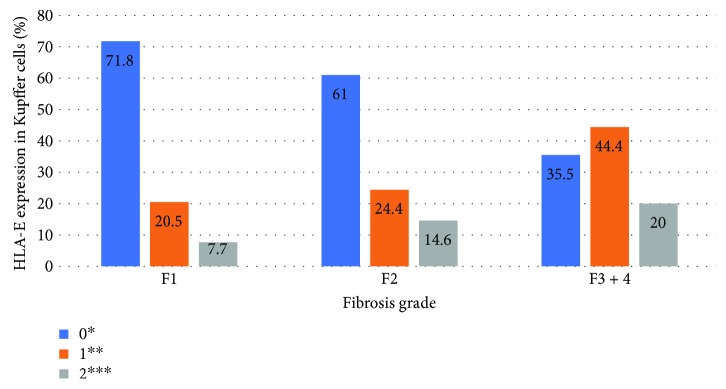
Association between HLA-E expression in Kupffer cells and liver fibrosis. HLA-E expression was described in the following grades: 0^∗^ (without positive cell expression), 1+^∗∗^ (<25%), 2+^∗∗∗^ (25–50%). There was no grade 3+ expression in this case. Fibrosis was described according to the METAVIR classification: F1: portal fibrosis without septa, F2: portal fibrosis with rare septa, F3-4: numerous septa without and with cirrhosis. *P* = 0.02.

**Figure 5:**
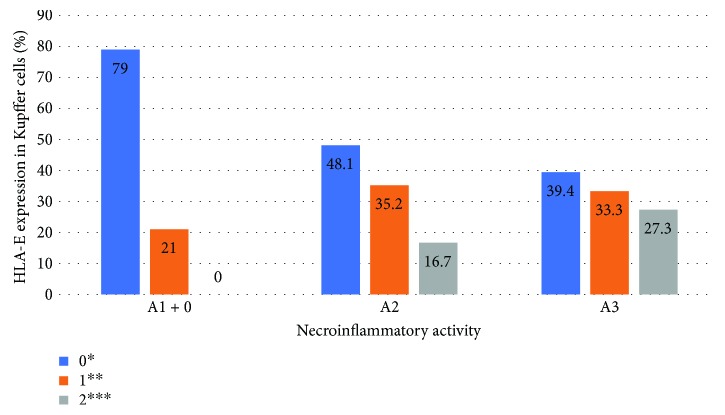
Association between HLA-E expression in Kupffer cells and liver necroinflammatory activity. HLA-E expression was described in the following grades: 0^∗^ (without positive cell expression), 1+^∗∗^ (<25%), 2+^∗∗∗^ (25–50%). There was no grade 3+ expression in this case. Necroinflammatory activity was described according to the METAVIR classification: A0 + 1: no or mild activity, A2: moderate activity, A3: severe activity. *P* = 0.002.

Significant association (*P* < 0.05) was found between degrees of necroinflammation and fibrosis and HLA-E expression in hepatocytes and Kupffer cells in HCV patients. The liver samples classified as severe fibrosis (METAVIR F3-F4) or necroinflammatory activity (METAVIR A3) presented greater expression of HLA-E in Kupffer cells and hepatocytes, with a linear association (*P* < 0.05). Figures 2–5 show the association between HLA-E expression and liver fibrosis/necroinflammatory activity.

## 4. Discussion

In this study, a significant upregulation of HLA-E expression was found in livers derived from HCV-infected individuals compared to HCV-negative subjects. Expression of HLA-E was detected on CD68(+) macrophages/Kupffer cells and hepatocytes. Nattermann et al. also found enhanced intrahepatic HLA-E expression in HCV-infected livers as compared to HCV-negative livers. Expression of HLA-E was detected in CD68(+) macrophages/Kupffer cells and CD31(+) sinusoidal endothelial cells, as well as in CD14(+) and CD83(+) cells [5]. They showed that, as a potential underlying mechanism, HCV gives rise to a peptide (HCV core amino acids 35–44) that binds to HLA-E, stabilizes its surface expression, and thereby impairs NK cell cytotoxicity [5, 14].

NK cells importantly contribute to the initial control of viral infections through their rapid and potent cytotoxic activity. They also exert an important antifibrotic activity on activated HSCs. Thus, an impaired function of NK cells can predispose the evolution to chronic hepatitis C infection and to the progression of liver fibrosis [5–10], the HLA-E molecule being a key component of this process via its interaction with inhibitory NK cell receptors. Nattermann et al. found a significantly increased proportion of inhibitory receptors, NKG2A, expressed in NK cells in HCV-positive patients, resulting in reduced cytolytic activity against cells incubated with the HLA-E-stabilizing peptide HCV core 35–44 [15]. Apart from NK cell inhibition, HLA-E upregulation may have further functional consequences. Increased HLA-E expression was seen particularly on hepatic CD68(+) macrophages/Kupffer cells, which should protect these cells against lysis by NK cells and thus prolong antigen presentation [5].

Epidemiological investigations have shown that fibrosis is accelerated in some situations, such as among older males [16]. Age is an independent factor, associated with a higher rate of fibrosis progression [17]. Studies have shown that the rate of progression can be 300 times greater in patients affected by the disease in their seventh decade of life compared to those affected in the third decade of life [18]. Similarly, male gender is also considered to be an important factor for the progression of hepatic fibrosis, increasing the rate of fibrosis up to 10 times, independent of age [18]. The aging of the immune system can also be related to these findings, since liver samples from HCV patients older than 40 years and male gender showed greater frequency of HLA-E expression on hepatocytes and Kupffer cells compared to younger female patients, as observed in the present study.

Liver fibrosis progression in HCV patients remains an important clinical issue, and it is a difficult phenotype to investigate. Since the precise date of HCV infection is often not available, it is confounded by environmental factors, which are difficult to quantify accurately, and there are limitations to the practicality and precision of longitudinal assessments [16].

We reported for the first time that HLA-E expression in the liver microenvironment was significantly higher in severe stages of fibrosis and necroinflammatory activity in HCV-infected patients. Persistent HCV infection is typically associated with chronic inflammatory changes within the liver, ultimately resulting in fibrosis and cirrhosis. Not only direct cytopathic effects but also immune-mediated mechanisms are likely to be involved in liver injury, and our data reinforces the participation of immune response in liver fibrosis progression. In our analyses, liver samples classified as severe fibrosis and necroinflammatory activity presented greater expression of HLA-E in Kupffer cells and hepatocytes, with a linear association.

## 5. Conclusions

The nonclassical HLA-E molecule might have an immunomodulatory effect and a possible role in the severity of liver disease in chronic hepatitis C. Knowledge of the expression profile of HLA-E may aid in the identification of HCV-infected patients with a worse prognosis and less favourable outcomes.

## Figures and Tables

**Table 1:** Frequency of HLA-E expression in hepatocyte cytoplasm and Kupffer cell in HCV patients and control subjects.

HLA-E expression in hepatocyte cytoplasm								HLA-E expression in Kupffer cell						
	0^∗^		1+^∗∗^		2-3+^∗∗∗∗^			0^∗^		1+^∗∗^		2+^∗∗∗^		
	*N* ^ȵ^	%	*N* ^ȵ^	%	*N* ^ȵ^	%		*N* ^ȵ^	%	*N* ^ȵ^	%	*N* ^ȵ^	%	
HCV (*n* = 125)	52	41.6	47	37.6	26	20.8	*P*	69	55.2	38	30.4	18	14.4	*P*
Control (*n* = 20)	16	80	04	20	0	0	0.004	18	90	02	10	0	0	0.01

*N* ^ȵ^: number of individuals; %: percentage; *P*: *P* value. Grades of HLA-E expression: 0^∗^ (without positive cell expression), 1+^∗∗^ (<25%), 2+^∗∗∗^ (25–50%), and 2-3+^∗∗∗∗^ (>25% or >50% of positive cell expression). There was no 3+ HLA-E expression in Kupffer cell.

## Data Availability

The data used to support the findings of this study can be requested from the author by email (rcaraujo@hcrp.usp.br).
